# Nrf2 Activators as Dietary Phytochemicals Against Oxidative Stress, Inflammation, and Mitochondrial Dysfunction in Autism Spectrum Disorders: A Systematic Review

**DOI:** 10.3389/fpsyt.2020.561998

**Published:** 2020-11-20

**Authors:** Jiaxin Yang, Xi Fu, Xiaoli Liao, Yamin Li

**Affiliations:** ^1^Clinical Nursing Teaching and Research Section, The Second Xiangya Hospital, Central South University, Changsha, China; ^2^Department of Xiangya Nursing School, Central South University, Changsha, China

**Keywords:** Nrf2 activator, Autism spectrum disorder, oxidative stress, inflammation, mitochondrial dysfunction, dietary supplements

## Abstract

Autism spectrum disorder (ASD) is a pervasive neurodevelopmental disorder with limited available treatments and diverse causes. In ASD patients, numerous researches demonstrated various alterations in inflammation/immune, oxidative stress, and mitochondrial dysfunction, and these alterations could be regulated by Nrf2. Hence, we aimed to systematically review the current evidence about the effects of Nrf2 activator supplementation on ASD objects from *in vitro* studies, animal studies, and clinical studies. Relevant articles were retrieved through searching for the Cochrane Library, PubMed, Web of Science, Scope, Embase, and CNKI databases (through September 23, 2020). Ultimately, we identified 22 preclinical studies, one cell culture study, and seven clinical studies, covering a total of five Nrf2 activators. For each Nrf2 activator, we focused on its definition, potential therapeutic mechanisms, latest research progress, research limitations, and future development directions. Our systematic review provided suggestive evidence that Nrf2 activators have a potentially beneficial role in improving autism-like behaviors and abnormal molecular alterations through oxidant stress, inflammation, and mitochondrial dysfunction. These dietary phytochemicals are considered to be relatively safer and effective for ASD treatment. However, there are few clinical studies to support the Nrf2 activators as dietary phytochemicals in ASD, even though several preclinical studies. Therefore, caution should be warranted in attempting to extrapolate their effects in human studies, and better design and more rigorous research are required before they can be determined as a therapeutic option.

## Introduction

Autism spectrum disorder (ASD) is a pervasive neurodevelopmental disorder characterized by impaired communication and social interaction, as well as repetitive/stereotyped behaviors (1, 2). The world health organization (WHO) reported in 2017 that one in 160 children worldwide has ASD (3), currently affected 1 in 54 children in the United States (4, 5), predominantly males (6). ASDs greatly limit the capacity of individuals to perform daily activities and participate in society, which imposes a significant emotional and economic burden on affected individuals and their families (3). Many factors may cause ASD, however, no definite pathogenesis have been established yet (5). There is currently no cure for ASD. Current drugs can only be used to control symptoms related to ASD, including anxiety, hyperactivity, epilepsy, obsessive behavior, and gastrointestinal problems (7).

Admittedly, numerous publications have indicated that the pathogenesis of ASD was affected by immune dysregulation/inflammation, oxidative stress, and mitochondrial dysfunction (8–10). Specifically, compared with normal children, children with ASD showed abnormal oxidative stress (i.e., glutathione (GSH) and the major intracellular antioxidant decreased, oxidized glutathione (GSSG) increased, and GSH/GSSG redox ratios decreased) in the peripheral and brain tissues (10, 11). Furthermore, the activity of several enzymes related to GSH synthesis and metabolic pathways in the brain tissue of ASD patients is reduced (11). Increasing researches have speculated that there may be an interaction between oxidative stress and mitochondrial function, which together affected the pathogenesis of ASD, although this remains somewhat controversial (11). Interestingly, the mitochondrial electron transport chain is not only a source of free radicals but also a target of free radicals. Consequently, oxidative stress may damage mitochondrial function, conversely, the abnormal mitochondrial function may cause further oxidative stress (12, 13). Moreover, biomarkers of mitochondrial dysfunction are associated with autistic behavior or severity (9). On the whole, mitochondrial dysfunction may contribute to ASD pathogenesis. Indeed, researchers found mitochondria dysfunction in different types of ASD objects, from ASD animal models, to cell lines (e.g., lymphocytes and granulocytes) derived from children with ASD (14, 15), to brain tissues of ASD patients (9, 16, 17). Finally, multiple studies evidenced inflammation/immune dysregulation in individuals with ASD both within the brain and the periphery. Specifically, researchers identified abnormal alterations in microglial cell activation, atypical pro-inflammatory cytokine production, immune-related gene expression, and other inflammatory biomarkers, where occurs in the central neural system (CNS) and peripheral immune system (18). Above all, those alterations may contribute to the pathophysiology of ASD.

**Figure 1:**
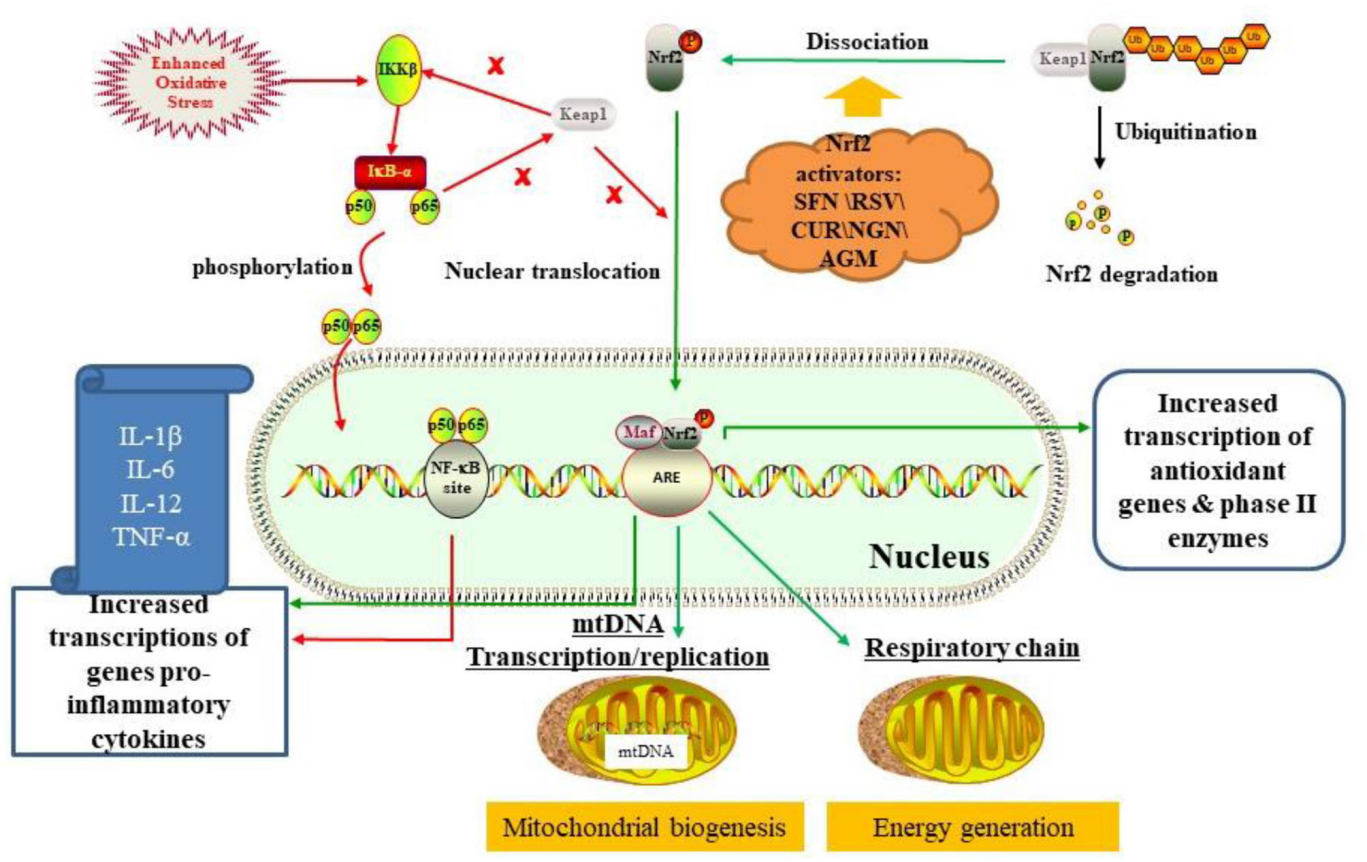
The mechanism of Nrf2 activation for ASD treatment by regulating oxidative stress, inflammation, and mitochondrial function by activating the Nrf2-ARE pathway (see main text). Besides, there is an interaction between the Nrf2/ARE pathway and the NF-κB pathway. On the one hand, free Keap1 prevents degradation of IkBα, thereby inhibiting NF-κB pathway. On the other hand, the p65 subunit of NF-κB also inhibits Keap1 from interfering with nuclear transcription of Nrf2. Dietary phytochemicals like sulforaphane (SFN), resveratrol (RSV), curcumin (CUR), naringenin (NGN), and agmatine (AGM) activated Nrf2/ARE pathway by interacting with Keap1.

Strikingly, existing evidence has indicated that Nrf2 plays an important role in immune dysregulation/inflammation, oxidative stress, and mitochondrial dysfunction. Inhibition of Nrf2 activity in human brains can increase the risk of chronic diseases such as Parkinson's disease (PD), Alzheimer's disease (AD), and amyotrophic lateral sclerosis (ALS) (19). Nrf2 is a transcription factor that regulates cellular oxidative stress responses. Under physiological conditions, Nrf2 is coupled with Kelch-like ECH-associated protein 1 (Keap1) and exists in the cytoplasm in an inactive form, which is then rapidly degraded by the ubiquitin-proteasome system, thereby maintaining stable expression of Nrf2 (20). Under pathological conditions, Nrf2 dissociates from Keap1, then translocated into the nucleus. In the nucleus, Nrf2 will bind to Maf protein to form a heterodimer, which interacts with antioxidant reaction elements (ARE) to regulate the expression of antioxidant proteins and phase II enzymes (21) (Figure 1).

Nuclear factor kappa-light-chain-enhancer of activated B cells (NF-κB) is a key regulator of inflammation (22). Under the physiological condition, NF-κB is coupled with an inhibitor of nuclear factor κ-B kinase subunit beta (IKK-β) protein and exits in the cytoplasm (23). Under pathological conditions (e.g., enhanced oxidative stress), IKKβ was activated, causing phosphorylation of NF-kappa-B inhibitor alpha (IkBα). Subsequently, NF-κB will migrate to the nucleus and bind to its region, which leads to transcription of pro-inflammatory cytokines such as IL-1β, IL-6, IL-12, and TNF-α (23, 24). Interestingly, there is an interaction between the Nrf2/ARE pathway and the NF-κB pathway. On the one hand, free Keap1 prevents degradation of IkBα, thereby inhibiting NF-κB pathway. On the other hand, the p65 subunit of NF-κB also inhibits Keap1 from interfering with the nuclear transcription of Nrf2 (24, 25) (Figure 1). Compared with normal animals, Nrf2-deficient mice have more pronounced effects on lipopolysaccharide (LPS)-induced microglial activation and inflammatory response, with lower levels of IL-1β, IL-6, IL-12, and TNF-α in the brain (23). Accordingly, the functional Nrf2 system is very important for regulating neuroinflammation and oxidative stress in the brain (19).

Moreover, increasing evidence indicated that Nrf2 may regulate mitochondrial function and metabolism. Studies have confirmed that Nrf2 deficiency can lead to mitochondrial depolarization, reduced ATP levels, and impaired respiratory function. On the contrary, the above negative phenomena can be reversed by activating Nrf2 genes (26, 27) (Figure 1). Therefore, Nrf2 can directly regulate cellular energy metabolism by regulating the availability of mitochondrial respiratory substrates.

Accordingly, Nrf2 activation may reverse the pathophysiological and behavioral abnormalities associated with ASD through the above-mentioned mechanisms. Interestingly, Nrf2 activator therapy for ASD has attracted widespread clinical interest. Several known Nrf2 natural activators (e.g., curcumin, sulforaphane, resveratrol, and naringenin) played an important role in regulating Nrf2 mechanisms (11, 28, 29). Thus, we systematically reviewed current evidence on the effects of Nrf2 activators administration on ASD objects from *in vitro* studies, animal studies, and clinical studies, aiming to elucidate molecular mechanisms related to the etiology of ASD and to open up a new approach to prevent and/or treat ASD.

## Materials and Methods

This systematic review was conducted and reported by the Preferred Reporting Items for Systematic Reviews and Meta-Analyses (PRISMA) guidelines (30).

### Search Strategy

We retrieved relevant articles in the Cochrane Library, PubMed, Web of Science, Scope, Embase, and CNKI up to September 23, 2020. We employed keywords combined with medical subject headings (MeSH) search, using the following terms: (Autism OR Autism Spectrum Disorder OR autistic OR autism disorder OR ASD OR Asperger Syndrome OR Pervasive Developmental Disorder) AND (Nuclear factor erythroid 2 related factors 2 activators OR NFE2 Related Factor 2 activator OR Nrf2 activator OR sulforaphane OR SFN OR broccoli OR Brussel sprouts OR watercress OR resveratrol OR curcumin OR naringenin OR agmatine). Additionally, we screened two registered clinical trial websites (i.e., clinicaltrials.gov and isrctn.com). Moreover, we manually searched the reference lists of the relevant articles to search for other articles.

### Eligibility Criteria

For the records, the following criteria were applied: (1) original research published in English and Chinese, (2) identifying data regarding Nrf2 activators therapy in animal or cell culture models of autism, or patients with ASD. Reviews, editorials, and commentaries were excluded. We also excluded meeting abstracts unless it reported available outcomes.

### Study Selection and Data Extraction

After removing duplicates, we filtered the titles and abstracts, and then the full texts. The following data was synthesized from each article: (a) literature information (author, publication year, country), (b) therapy type, (c) study design, (d) intervention details, (e) main findings, and (f) adverse effect. Given the exploratory status in this field, maybe it is justified not to report the methodological quality of these studies.

## Result

### Overview of the Included Studies

**Figure 2:**
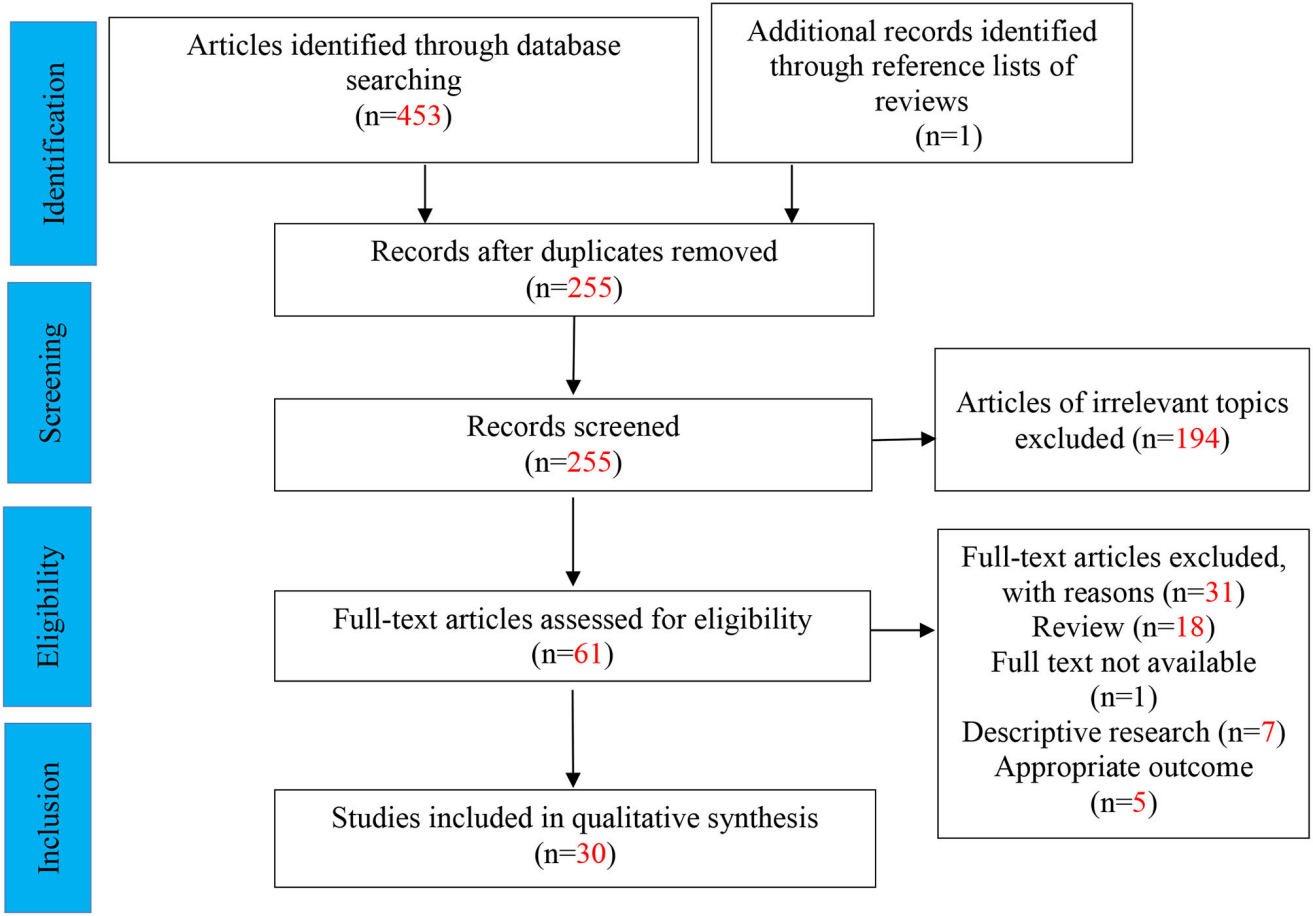
Flow diagram for selection of studies (PRISMA flow diagram).

We initially yielded 453 articles, 30 of which were ultimately eligible for the systematic review (Figure 2). Of all 30 articles, 7 represented human clinical trials, 22 represented animal studies, and one represented cell culture study. Of these, five Nrf2 activators related to ASD treatment were studied (i.e., sulforaphane, resveratrol, naringenin, curcumin, and agmatine), varying different intervention dosage and duration. Overall, these studies provided evidence that various types of Nrf2 activators may have potential beneficial and protective effects on autism-like behaviors and abnormal molecular alterations, although with divergence. Amongst 22 animal studies, fifteen and five studies were performed in rat and mice models, respectively. Animal models of ASD include pharmacologic model and genetically modified animal models, such as BTBR T~(+)tf/J (BTBR) model (*n* = 6), prenatal exposure to valproic acid (VPA) (*n* = 12), intracerebroventricular administration of propanoic acid (PPA) (*n* = 3), and prenatal exposure to progestins (*n* = 1). Of the 7 clinical studies, four were randomized controlled trials (RCTs), two were open-label trials, and one was a follow-up case series. Participants were mostly male aged 3–30 years old. The key characteristics of the included studies were shown in Tables 1, 2.

**Table 1:** Behavioral and molecular effects of Nrf2 activators in animal and cell culture models of ASD.

**Study**	**Therapy**	**Animal model/Analysis object**	**Interventional details**	**Main findings**
Juybari et al. (31)	RSV	Prenatal exposure to VPA in Wistar rats Male and female offspring	On E6.5 the pregnant rats were randomly separated into 4 groups: (1) VPA: a single i.p. injection of 600 mg/kg VPA at E12.5. (2) RSV: injected with RSV (3.6 mg/kg, i.p.) (3) RSV + VPA: RSV injection plus VPA injection (4) Vehicle: saline or DMSO injection Intervention duration: 13 days (E6.5 to E18.5)	Prenatal RSV treatment alleviated social deficits
Hidema et al. (32)	RSV	Prenatal exposure to VPA in WT mice Male offspring	On E13.5 the mice were randomly separated into 6 groups: (1) Control: injected with corn oil by i.p. (2) RSV: injected with RSV (30 mg/kg, i.p.) (3) *Oxtr*-KO: injected with corn oil by i.p. (4) *Oxtr*-KO + RSV: injected with RSV (30 mg/kg, i.p.) (5) VPA + RSV: a single i.p. injection of 600 mg/kg VPA at E13.5 plus injection with RSV (6) VPA + corn oil: RSV injection plus VPA injection	A single administration of RSV in male *Oxtr*-KO mice and VPA-ASD mice can improve social impairments, and up-regulated Sirt1 gene and Egr3 gene expressions in the amygdala of the *Oxtr*-KO mice
Fontes-Dutra et al. (33)	RSV	Prenatal exposure to VPA in Wistar rats Male offspring	On E6.5 the pregnant rats were randomly separated into 4 groups: (1) Control: DMSO injection; (2) RSV: injected with RSV (3.6 mg/kg, i.p.) plus DMSO; (3) VPA: a single i.p. injection of 600 mg/kg VPA at E12.5 plus DMSO; (4) RSV + VPA: RSV injection plus VPA injection Intervention duration: 13 days (E6.5 to E18.5)	No significant effect was found in empathy-like behaviors
Fontes-Dutra et al. (34)	RSV	Prenatal exposure to VPA in Wistar rats Male and female offspring	On E6.5 the pregnant rats were randomly separated into 4 groups: (1) Control: DMSO injection; (2) RSV: injected with RSV (3.6 mg/kg, i.p.) plus DMSO; (3) VPA: a single i.p. injection of 600 mg/kg VPA at E12.5 plus DMSO; (4) RSV + VPA: RSV injection plus VPA injection Intervention duration: 13 days (E6.5 to E18.5).	The prenatal treatment of RSV improved sensory deficits (assessed by Nest Seeking Behavior and Whisker Nuisance Task); RSV promoted nervous system development, regulated neuronal proliferation, migration, and the establishment of synaptic connections
Ahmad et al. (35)	RSV	BTBR model Male mice aged 6–8 weeks	Mice were randomly separated into 2 groups: (1) injected with DMSO (i.p.); (2) injected with RSV (40 mg/kg RSV, i.p.) Intervention duration: 7 days	RSV decreased mRNA and protein expression levels of TLR2, TLR3, TLR4, NF-κB, iNOS, and COX-2 in brain tissue, and CD4+TLR2+, CD4+TLR3+, CD4+TLR4+ CD4+NF-κB+, and CD4+iNOS+ levels in spleen cells
Hirsch et al. (36)	RSV	Prenatal exposure to VPA in Wistar rats Male offspring	On E6.5 the pregnant rats were randomly separated into 4 groups: (1) Control: DMSO injection; (2) VPA: a single i.p. injection of 600 mg/kg VPA plus DMSO; (3) RSV: injected with RSV (3.6 mg/kg) plus DMSO; (4) RSV + VPA: RSV injection plus VPA injection Intervention duration: 13 days (E6.5 to E18.5)	RSV alleviated social deficits but not the symptoms of repetitive behaviors (assessed by three-chambered and self-grooming test); RSV upregulated the level of miR134-5p in VPA mice
Ahmad et al. (37)	RSV	BTBR model Male mice aged 6–8 weeks	Mice were randomly separated into 3 groups: (1) injected with DMSO (i.p.); (2) injected with RSV (20 mg/kg RSV, i.p.); (3) injected with RSV (40 mg/kg RSV, i.p.). Intervention duration: 7 days	RSV treatment attenuated cytokine expression (i.e., IL-6, TNF-α, IFN-γ), and down-regulated JAK1-STAT3 transcription factors signaling
Dai et al. (38)	RSV	Prenatal exposure to VPA in Wistar rats Male offspring	On E6 the pregnant rats were randomly separated into 4 groups: (1) Control: DMSO injection; (2) RSV: injected with RSV (3.6 mg/kg, i.p.) plus DSMO; (3) VPA: a single i.p. injection of 600 mg/kg VPA plus DSMO; (4) RSV + VPA: RSV injection plus VPA injection Intervention duration: 13 days	RSV attenuated VPA-triggered down-regulation of SIRT1 and LC3-II in the hippocampus, prefrontal cortex, and cerebellum; RSV corrected the VPA-induced autism-like behaviors
Bakheet et al. (39)	RSV	BTBR model Male mice aged 6–8 weeks	Mice were randomly separated into three groups: (1) injected with DMSO (i.p.); (2) injected with RSV (20 mg/kg RSV, i.p.); (3) injected with RSV (40 mg/kg RSV, i.p.); Intervention duration: 7 days	RSV improved the symptoms of repetitive behaviors; RSV modulated the levels of Th1, Th2, Th17, and T regulatory cell-related transcription factor (i.e., RORγt, IL-17A, GATA-3, T-bet, and Foxp3)
Bhandari and Kuhad (40)	RSV	PPA infused into ICV in SD rats Male rats aged 3–4 months	Rats were randomly separated into 5 groups: (1) Control: injected with PBS (ICV); (2) PPA: injected with PPA (ICV); (3) PPA+RSV: PPA plus RSV (5 mg/kg/day, peroral); (4) PPA+RSV: PPA plus RSV (10 mg/kg/day, peroral); (5) PPA+RSV: PPA plus RSV (15 mg/kg/day, peroral) Intervention duration: 28 days	RSV significantly and dose-dependently improved core symptoms of ASD; RSV reduced oxidative stress markers (lipid hydroperoxide and nitrite) and increased the endogenous antioxidant (GSH, superoxide dismutase, and catalase) in the brain RSV significantly and dose-dependently attenuated MMP-9 and TNF-α levels
Xie et al. (41)	RSV	Prenatal exposure to different progestins in SD rats Male and female offspring	**(a) Postnatal treatment protocol:** (1) VEH/Control: pregnant injected with VEH [subcutaneous injection at the nape, E1 to pub delivery) plus control (offspring received carboxymethylcellulose (10 ml/kg, orally for 4 weeks)]; (2) NET/Control: pregnant injected with NET (20 mg, subcutaneous injection at the nape, E1 to pub delivery) plus control; (3) VEH/RSV: VEH plus RSV [offspring received RSV (20 ml/kg, orally for 4 weeks)] (4) NET/RSV: NET plus RSV **(b) Prenatal treatment protocol: the pregnant rats were randomly separated into 4 groups at E1 (Intervention duration: from E1 to pub delivery):** (1) VEH/Control: injected with VEH (subcutaneous injection at the nape) plus control (10 ml/kg of 10 g/l carboxymethylcellulose, orally); (2) NET/Control: injected with progestin (20 μg, subcutaneous injection at the nape) plus control; (3) VEH/RSV: VEH plus RSV (20 mg/kg, orally) (4) NET/RSV: NET plus RSV	Postnatal and prenatal RSV treatment, respectively, alleviated ERβ suppression and autism-like behaviors; Postnatal RSV treatment ameliorated oxidative stress and modulated mitochondrial function and lipid metabolism
Bakheet et al. (42)	RSV	BTBR model Male mice aged 6–8 weeks	Mice were randomly separated into 3 groups: (1) injected with DMSO (i.p.); (2) injected with RSV (20 mg/kg RSV, i.p.); (3) injected with RSV (40 mg/kg RSV, i.p.); Intervention duration: 7 days	RSV inhibited chemokine receptor expression (i.e., CCR3, CCR5, CCR7, CCR9, CXCR3, and CXCR5) in the brain and spleen
Bambini-Junior et al. (43)	RSV	Prenatal exposure to VPA in Wistar rats Male offspring	On E6.5 the pregnant rats were randomly separated into 4 groups: (1) Control: DMSO injection; (2) RSV: injected with RSV (3.6 mg/kg, i.p.) plus DMSO; (3) VPA: a single i.p. of 600 mg/kg VPA plus DMSO; (4) RSV + VPA: RSV injection plus VPA injection Intervention duration: 13 days (E6.5 to E18.5)	RSV prevents autism-like social behaviors (three-chambered test);
Nadeem et al. (44)	SFN	BTBR model Male mice aged 8–10 weeks	Mice were randomly separated into 2 groups: (1) injected with vehicle, i.p.; (2) injected with SFN (50 mg/kg, i.p.) Intervention duration: 7 days	SFN improved social defects and repetitive behaviors (assessed by marble-burying test and three-chambered sociability test); SFN reduced Th17 related immune responses such as STAT3, RORC, IL-17 A, and IL-23R at both mRNA and protein expression level; SFN reduced oxidative stress (p-NFκBp65, iNOS, and nitrotyrosine), and upregulates enzymatic antioxidants in the periphery and cerebellum
Nadeem et al. (45)	SFN	Cell culture with LPS **Cell from human PBMCs and monocytes** 38 ASD (32M/ 6F)	PBMCs/monocytes were incubated overnight with or without SFN (5 μM final concentration) in the presence/absence of LPS (1 μg/ml, final concentration) in 48-well-culture plates	SFN augmented enzymatic antioxidants and decreased inflammation responses and nitrative stress in monocytes of ASD subjects
Liu et al. (46)	SFN	Cell culture with LPS Cell from healthy adults' PBMCs TD	PBMCs were treated with VEH PBMCs were treated with SFN (2 μM) for 6 h PBMCs were treated with SFN (5 μM) for 6 h PBMCs were exposed to VEH repeatedly for 3 consecutive days PBMCs were exposed to VEH repeatedly for 3 consecutive days plus LPS (1 ng/ml) for 5 h PBMCs were exposed to SFN (0.5 μM) repeatedly for 3 consecutive days plus LPS (1 ng/ml) for 5 h	SFN induced cytoprotective gene expression and decreased pro-inflammatory gene expression
Zhong et al. (47)	CUR	BTBR model Male mice aged 6 days	Mice were randomly separated into 4 groups: (1) C57+DMSO (2) C57+CUR (20 mg/kg, i.p.) (3) BTBR+DMSO (4) BTBR+CUR (20 mg/kg, i.p.) (5) Intervention duration: 3 days (P6 to P8)	Neonatal CUR treatment improved autism-related symptoms in BTBR mice (i.e., enhancing sociability, reducing repetitive behaviors, and ameliorating cognitive impairments), and rescued the suppression of hippocampal neurogenesis in BTBR mice
Huang (48)^a^	CUR	Prenatal exposure to VPA in Wistar rats **Male and female offspring**	VPA-pubs were randomly separated into 2 groups: (1) injected with PBS (i.p.,) plus DMSO; (2) injected with CUR (50 mg/kg, i.p.) plus DMSO+PBS Intervention duration: 3 weeks (starting the 7th after birth)	CUR improved the abnormal development and behavior, up-regulated the expression of neurons in hippocampal DG and CA3 regions, and down-regulated the expression of astrocytes of autistic model rats
Al-Askar et al. (49)	CUR	Prenatal exposure to VPA in Wistar rats **Male offspring**	Male VPA-pubs were randomly separated into 2 groups: (1) **Control:** received saline (orally) at day 7 after birth; (2) **(****2****)** CUR: received CUR (1 g/kg, orally) orally at 7 days after birth	CUR moderately corrected dysfunctions in some parameters (i.e., IFN-γ, serotonin, glutamine, GSH, GSH S-transferase, lipid peroxidase, CYP450, IL-6, glutamate, and GSSG), and improved delayed maturation and abnormal weight
Chen et al. (50)	CUR	Prenatal exposure to VPA in Wistar rats **Male and female offspring**	VPA-Pubs were randomly separated into 2 groups: (1) pubs received an i.p injection of PBS with 1 mL/L DMSO; (2) pubs received an i.p. injection of 50 mg/kg CUR (1mL/L DMSO in PBS) The treatment lasted 14 days	CUR increased the number of social behaviors, decreased latency to social behavior, and reduced repetitive behavior; CUR increased BDNF integral optical density values of autism rats in the temporal cortex
Bhandari and Kuhad (51)	CUR	PPA infused into ICV in SD rats **Male rats aged 3–4 months**	Rats were randomly separated into 8 groups: (1) naïve control animals; (2) ICV injection of sodium acetate control (4.0 μl of 1 M solution in PBS); (3) ICV injection of PBS (4.0 μl); (4) ICV injection of 4.0 μl of 1 M propanol in PBS; (5) ICV injection of 1 M PPA (4.0 μl in PBS); (6) ICV injection of PPA + CUR (50 mg/kg/day; peroral); (7) ICV injection of PPA +CUR (100 mg/kg/day; peroral); (8) ICV injection of PPA +CUR (200 mg/kg/day; peroral); Intervention duration: 26 days (from the 3rd day to the 28th day after surgery)	CUR significantly and dose-dependently restored abnormalities in the neurology, behaviors, and molecular biology
Chen (52)^a^	CUR	Prenatal exposure to VPA in Wistar rats **Male and female offspring**	VPA-pubs were randomly separated into 4 groups: (1) injected with PBS (i.p.); (2) injected with CUR (10 mg/kg, i.p.); (3) injected with CUR (30 mg/kg, i.p.); (4) injected with CUR (50 mg/kg, i.p.); Intervention duration: 14 days	The low dose of CUR had no significant effect on autistic behavior and BDNF expression; the medium dose could partially improve autistic behaviors, but it had no significant effect on BDNF expression; the large dose had the most significant effect on improving autistic behaviors and on BDNF expression in the temporal cortex
Bhandari et al. (53)	NGN	PPA infused into ICV in SD **Male rats aged 3-4 months**	Rats were randomly separated into 10 groups: (1) naïve control animals; (2) PPA; (3) PPA + NGN (25 mg/kg); (4) PPA + NGN (50 mg/kg); (5) PPA + NGN (100 mg/kg); (6) PPA + minocycline (50 mg/kg); (7) blank -PLGA-NP; (8) PPA + NGN-PLGA-NP (25 mg/kg); (9) PPA + GLU-NGN-PLGA-NP (25 mg/kg); (10) PPA + Tween 80-NGN-PLGA-NP (25 mg/kg) Intervention duration: 29 days (thrice daily, orally)	NGN and its NPs significantly restored autism-like behaviors and abnormal molecular alterations GSH and tween 80 coated NPs strengthened brain delivery of NGN by inhibiting P-glycoprotein NGN (100 mg/kg) and its NPs (25 mg/kg) showed comparable pharmacological efficacy to minocycline (50 mg/kg)
Kim et al. (54)	AGM	Prenatal exposure to VPA in Sprague-Dawley rats **Male offspring**	Male VPA-pubs were randomly separated into 5 groups: (1) Pubs received saline, i.p.; (2) pubs received 25 mg/kg AGM, i.p.; (3) pubs received 50 mg/kg AGM, i.p.; (4) pubs received 100 mg/kg AGM, i.p. The treatment conducted 30 min before each behavior test (5) pubs received an inhibitor of AGM with 50 mg/kg before AGM treatment.	AGM (over 50 mg/kg), and not its metabolites, improved social defects, repetitive and hyperactive behaviors; AGM normalized overactive ERK signals in the prefrontal cortex and hippocampus of VPA-exposed rat

ASD, autism spectrum disorder; VPA, valproic acid; PPA, propanoic acid; ICV, intracerebroventricular; SD, Sprague Dawley; VEH, vehicle; i.p: intraperitoneal; PBS, phosphate buffer saline; E, embryonic day; NET, norethindrone; DMSO, Dimethyl sulfoxide; SFN, sulforaphane; RSV, resveratrol; NGN, naringenin; AGM, agmatine; CUR, curcumin; COX, Cyclooxygenase; Inos, Inducible nitric oxide synthase; TLR, Toll-like receptors; MMP-9, matrix metalloproteinases; PLGA, poly lactic-co-glycolic acid; NP, nanoparticles; GSH, glutathione; GSSG, oxidized glutathione; iNOS, inducible nitric oxide synthase; SOD, superoxide dismutase; GPx, glutathione peroxidase; JAK-STAT, Janus kinase/signal transducers and activators of transcription; IL, interleukin; TNF, tumor necrosis factor; INF, interferon; CCR, chemokine receptor; Th, T helper; LPS, lipopolysaccharide; ERK, extracellular signal-regulated kinase; BNDF, brain-derived neurotrophic factor;

a *master thesis; WT, Wilde-type C57BL6/J (WT) and Wild-type DBA/2; Oxtr-KO: oxytocin receptor gene knockout; Sirt1, silent information regulator 1; Egr3, early growth response factor 3*.

**Table 2:** Clinical studies with Nrf2 activators in patients with ASD.

**Study/Country**	**Therapy**	**Study design**	**Interventional details**	**Main findings**	**AEs**
Hendouei et al. (55) Iran	RSV	A double-blind, placebo-controlled randomized clinical trial **E:** 31 (25 M/6F) **P:** 31 (25 M/6F) 4–12 years	**E:** risperidone + RSV **P:** risperidone + placebo **Risperidone dosage:** starting at a dose of 0.5 mg twice daily with a dose increase of 0.5 mg per week (for the first 3 weeks) **RSV dosage:** 250 mg twice per day for 10 weeks	No significant effect for adjunctive treatment with RSV on irritability but hyperactivity/non-compliance of ASD patients	The most frequent AEs were restlessness, constipation, and diarrhea in the resveratrol group and restlessness, increased appetite, and constipation in the placebo group The AEs of treatment with two groups were comparable
Momtazmanesh et al. (56) Iran	SFN	A randomized, double-blind, placebo-controlled clinical trial E: 30 (19 M/11F) P: 30 (21 M/9F)	**E:** risperidone + SFN **P:** risperidone + placebo **Risperidone dosage**: started at a dose of 0.25 mg (weighing <20 kg) or 0.5 mg (weighing ≥20 kg), increased stepwise by 0.5 mg weekly up to a maximum dose of 1 mg (weighing <20 kg), 2.5 mg (weighing 20–45 kg), and 3.5 mg (weighing >45 kg). **SFN dosage**: 50 μmol and 100 μmol daily for patients weighing <45 kg and 45–90 kg, respectively	The results supported the efficacy of SFN as an adjuvant to risperidone for improvement of irritability and hyperactivity symptoms in children with ASD	No severe AEs AEs: increased appetite, headache, and diarrhea
Lynch et al. (57) America	SFN	A follow-up case series 16 ASD	SFN-rich broccoli sprout extract was administered once a day orally **Dose:** <101 lbs: ~ 50μmol; 101–199 lbs: ~ 100 μmol; >199 lbs: ~ 150 μmol The study lasted for 3 years	SFN has beneficial effects both during the SFN intervention phase and the subsequent three-year follow-up period	SFN was safe and well-tolerated
Singh et al. (58)^a^ America	SFN	A randomized, double-blind, placebo-controlled phase-2 clinical trial 50 ASD 3–12 years	SFN (contains 125 mg broccoli seed powder/tablet) were administered once a day orally **Dose:** 30–50 lb: 45 μmol/day; 50–70 lb: 60 μmol/day; 70–90 lb: 90 μmol/day; 90–110 lb: 105 μmol/day; 110–130 lb: 120 μmol/day The trial lasted 36 weeks	SFN appears to be safe and effective in children with ASD A preliminary analysis of the OACIS showed: 23% of patients had a great improvement at 7 weeks, 31% at 15 weeks, 59% at 22 weeks, and 53% at 30 weeks	Most AEs so far were mild and transient: insomnia (28%), vomiting (19%), flatulence (17%), diarrhea (15%), and constipation (13%)
Bent et al. (59) America	SFN	Open-label study 15 ASD (12 M/3F) 5–22 years	Avmacol, an SFN-producing dietary supplement **Dose:** 32–41 kg: 222 μmol GR/day, 41–50 kg: 259 μmol GR/day, 50–59 kg: 296 μmol GR/day, 59–68 kg: 333μmol GR/day, 68–77 kg: 370 μmol GR/day, 77–86 kg: 444 μmol GR/day, 86–95 kg: 481 μmol GR/day, and 95–105 kg: 555 μmol GR/day Once a day in the morning for 12 weeks	SFN improved the ABC score and significantly improved the symptom of social responsiveness (assessed by SRS);77 urinary metabolites related to oxidative stress, amino acid/gut microbiome, neurotransmitters, hormones, and sphingomyelin metabolism were correlated with ASD symptoms	Six families reported AEs which was unlikely to be related to the study supplement
Singh et al. (58) America	SFN	A placebo-controlled, double-blind, randomized trial **E:** 26 (M) **P:** 14 (M) 13–30 years	SFN-rich broccoli sprout extract was administered once a day orally **Dose:** <101 lbs: ~ 50 μmol; 101–199 lbs: ~ 100 μmol; >199 lbs: ~ 150μmol Intervention duration: 18 weeks followed by a 4-week washout period	It was observed that SFN treatment substantially ameliorated the core symptoms of ASD (assessed by ABC, SRS, AND CGI-I)	SFN was safe and well-tolerated There was no difference in AEs between the placebo group and the SFN group

*SFN, sulforaphane; RSV, resveratrol; M, male; F, female; ASD, autism spectrum disorder; PBMCs, peripheral blood mononuclear cells; AEs, adverse effect; OACIS, Ohio Autism Clinical Global Impressions Scale; ABC, Aberrant Behavior Checklist; SRS, Social Responsiveness Scale; CGI-I, Clinical Global Impression Improvement Scale; E, experiment; P, placebo; a, Conference abstract*.

### Application of Nrf2 Activators in ASD

Several Nrf2 activators showed improvements in autism-like behaviors and abnormal molecular alterations in clinical and preclinical studies.

### Resveratrol

Resveratrol (3,5,4′-Trihydroxystilbene, RSV) is a natural polyphenolic stilbenoid present in berries, nuts, red wine, and grapes. It is produced when attacked by bacteria, fungi, ultraviolet radiation, and chemical substances (60). As an agonist of Nrf2, RSV could increase the level of Nrf2 and induce its translocation into the nucleus, thereby activating genes with AREs (23), which ultimately increased the expression of various antioxidant enzymes and inflammatory cytokines. Recently, studies have shown that RSV has a neuroprotective role in various diseases, including neurodegeneration diseases, autoimmune disorders, heart diseases, and cancer (55, 61).

Thirteen studies investigated the effects of RSV treatment on animal models of ASD (Table 1). Prenatal RSV treatment [at doses of 3.6 mg/kg for 13 days, intraperitoneal (i.p.)] had different effects on VPA-induced autistic behaviors in six studies. On the one hand, four studies demonstrated that prenatal RSV treatment could reverse VPA-induced social deficits (31, 36, 38, 43). Moreover, RSV also prevented sensory deficits triggered by VPA (34). However, prenatal RSV can prevent some but not all abnormalities induced by VPA (34), which RSV counteracted the deficit in reduced total reciprocal social interaction but not in the repetitive behavior in the study by Hirsch et al. (36). On the contrary, Fontes-Dutra et al. (33) demonstrated that prenatal RSV treatment did not affect the deficits of empathy-like behavior in VPA animals. Other than prenatal RSV treatment, Hidema et al. demonstrated that postnatal RSV treatment (30 mg/kg, a single administration, i.p.) could improve social impairments induced by VPA (32). Besides, prenatal RSV treatment (20 or 40 mg/kg for 7 days, i.p.) significantly reduced repetitive behavior in BTBR mice, and the high-dose group was more significant (39). In the PPA model, RSV supplementation (5, 10, 15 mg/kg for 28 days, peroral) significantly and dose-dependently improved core symptoms of ASD (i.e., repetitive behavior and social interaction) as well as counteracting other abnormities (i.e., hyperactivity, anxiety, spatial learning, memory, and depression-like behaviors) (40). As for models of prenatal/postnatal exposure to progestins, oral administration of either postnatal or prenatal RSV treatment (at the dose of 20 mg/kg for 28 days) ameliorated autism-like behaviors (41). Accordingly, the timing, method, and dosage of RSV administration may have different effects on the autism-like behaviors induced by the same or different ASD models.

RSV prevented or ameliorated abnormalities in animal models of ASD through various molecular mechanisms, mainly anti-oxidative stress, and anti-inflammatory functions. Four studies investigated the effect of RSV treatment in the BTBR model, which was mediated by an anti-inflammatory mechanism (35, 37, 39, 42). Bakheet et al. (39, 42) elucidated that RSV treatment (20 and 40 mg/kg 7 days, i.p.) corrected dysfunction of inflammation-related transcription factor signaling. On the one hand, RSV treatment downregulated the mRNA expression of chemokine receptors (CCR) (i.e., CCR3, CCR5, CCR7, CCR9, CXCR3, and CXCR5) in the brain and spleen tissues, and decreased the levels of chemokine receptor in CD4+ T cells and spleen (42). On the other hand, RSV treatment ameliorated dysregulation of T helper (Th)1, Th2, Th17, and T regulatory cell-associated transcription factors in BTBR mice (39). By using flow cytometric, Real-Time PCR, and western blot analysis, we found that RSV decreased both mRNA and protein expression of T-bet, GATA-3, RORγt, and IL-17A, and upregulated Foxp3 expression in the spleen and brain tissues (39). Similarly, Ahmad et al. suggested that RSV treatment improved neuroimmune dysregulation by inhibiting pro-inflammatory mediators, TLRs/NF-κB transcription factor signaling, and JAK-1/STAT3 signaling in BTBR mice (35, 37). Specifically, RSV treatment not only significantly reduced the levels of CD4+TLR2+, CD4+TLR3+, CD4+TLR4+, CD4+NF-κB+, and CD4+iNOS+ in splenocytes (35), but also reduced the levels of TLR2, TLR3, TLR4, NF-κB, iNOS, and COX-2 mRNA and protein expression in brain tissues (35). Moreover, RSV significantly decreased IL-6+, TNF-α+, IFN-γ+, and Signal Transducer and Activator of Transcription (STAT)3+ in CD4+ spleen cells and decreased IL-6, TNF-α, IFN-γ, JAK1, and STAT3 mRNA expression levels as well as their protein expression level. In the PPA model, RSV supplementation (5, 10, 15 mg/kg) dose-dependently attenuated inflammation and oxidative stress (40). On the one hand, RSV reduced oxidative stress markers (lipid hydroperoxide and nitrite) and increased the endogenous antioxidants (GSH, superoxide dismutase, and catalase) in the brain. On the other hand, RSV normalized matrix metalloproteinases-9 levels associated with neuroinflammation and improved the activity of brain mitochondrial complex enzymes (40). MicroRNA (miRNA) is a small non-coding RNA regulating gene expression and associated with biological processes affected by neurodevelopmental disorders. Interestingly, Hirsch et al. demonstrated that RSV downregulated miR134-5p expression in VPA rats (36). Similarly, Fontes-Dutra et al. demonstrated that VPA altered the localization of PV^+^-neurons in the primary sensory cortex and amygdala, and reduced the level of gephyrin in the primary somatosensory area (34). However, prenatal RSV treatment prevented all the alterations induced by VPA (34). Prenatal exposure to progestins reduced estrogen receptor (ERβ) expression in the amygdala in the offspring, however, it can be reversed by postnatal or prenatal RSV treatment (41). Moreover, further research indicated that RSV activated ERβ and its target genes by demethylating the DNA and histones on the ERβ promoter, and then minimized oxidative stress, mitochondria dysfunction, and abnormal lipid metabolism in the brain (41). In conclusion, RSV has a potential impact on the prevention and/or treatment of ASDs.

Clinical research on RSV was still in its infancy. We only retrieved one RCT that assessed the potential therapeutic effects of RSV as an adjunct to risperidone on the irritability of ASD patients (55). Except for the symptom of hyperactivity/non-compliance, adjunctive treatment with RSV had no significant effect on most symptoms of ASD (i.e., irritability, lethargy/social withdrawal, stereotypic behavior, and inappropriate speech). Notably, ASD patients often comorbid ADHD with a prevalence of 26–70% (62). So, RSV plus risperidone treatment may play a beneficial role in ASD patients who comorbid ADHD. Thus, further RCTs could be devoted to studying the effect of RSV as monotherapy on certain subgroups of ASD. Lastly, this clinical study demonstrated no significant difference in the number and severity of adverse events between the two groups (55). Taken together, our findings revealed an important preventive role of RSV, ranging from autism-like behaviors to molecular alterations related to ASD. Further exploration of the therapeutic mechanisms of RSV could provide clues to reveal the biomarkers and etiology of ASD.

### Sulforaphane

Sulforaphane (1-isothiocyanate-4-methylsulfinyl butane, SFN) is an active compound extracted from cruciferous vegetables like broccoli, brussels sprouts, and cabbages (62, 63). Pharmacokinetic studies revealed that SFN concentration in human plasma increased rapidly after broccoli supplementation, reaching a peak several hours later (64). After being absorbed, SFN can easily cross the blood-brain barrier and quickly reach the central neuron system, exerting its neuroprotective effects (65, 66). Interestingly, SFN has a unique ability in Nrf2 activation, which enhances the expression of heme oxygenase-1 (HO-1) and nicotinamide adenine dinucleotide phosphate quinone oxidoreductase 1 (NQO1) (59, 67), thereby protecting neurons from 6-hydroxydopamine (6-OHDA), methyl-4-phenyl-1,2,3,6-tetrahydropyridine (MPTP) (68), amyloid β (Aβ) (69), and pro-inflammatory cytokines (70, 71). Moreover, in Nrf2-deficient mice, SFN has no significant effect on the pro-inflammatory response, indicating SFN might be an Nrf2-dependent anti-inflammatory agent (72, 73). Therefore, we speculate that Nrf2 activation mediates the anti-oxidant stress and anti-inflammatory effects of SFN, thereby exerting SFN's neuroprotective effect (73).

Six studies investigated the efficacy of SFN on autism-like behaviors, one of which was an animal study and others were human studies (Tables 1, 2). In the animal study, BTBR mice treated with SFN (at a dose of 50 mg/kg for 7 days, i.p.) had reduced repetitive behaviors, and increased social interaction in three-chambered sociability test compared to untreated. Other than in the animal study, SFN with various dosages has also shown potential effects in human studies (58). In the study by Singh et al., patients received SFN at a dose of 50–150 μmol/day for 18 weeks, followed by a 4-week washout period. It was observed that SFN treatment substantially ameliorated the core symptoms of ASD (58). Furthermore, researchers followed up and re-evaluated the cases from the study by Singh et al. Many parents and caregivers (57). Specifically, among follow-up cases, one case showed maintained behavioral improvements even after discontinuation of SFN, and nine cases were still taking SFN with persistent improvement (57). In an open-label study, SFN improved the ABC score and significantly improved the symptom of social responsiveness (assessed by SRS) (74). Furthermore, in a recent clinical trial (NCT02561481), a preliminary analysis of the Ohio Autism Clinical Impressions Scale (OACIS) showed that SFN appeared to be safe and effective in children with ASD. Besides, SFN not only had potential effects on partial autism-like behaviors as a monotherapy but also improved irritability and hyperactivity symptoms as an adjunct to risperidone (56).

Notably, five studies explored the impacts of SFN on molecular alterations caused by ASD. In the animal study, SFN-treated BTBR mice reduced Th17 immune responses (STAT3, RORC, IL-17 A, and IL-23R expression in CD4+ T cells) as well as oxidative stress parameters in neutrophils/cerebellum (NF-κB, iNOS, and lipid peroxides) (44). Furthermore, SFN upregulated enzymatic antioxidant defenses (44), which was consistent with the results of *in vitro* study (45). Nrf2 protein expression and binding activity were impaired in human PBMCs which cultured with LPS. However, SFN can activate Nrf2 with *ex vivo* treatment by reducing the NF-κB signaling, thereby reversing LPS-induced inflammation and nitrative stress in PBMCs (45). Liu et al. also obtained a similar result that SFN with *ex vivo* treatment decreased pro-inflammatory gene expression induced by LPS in human PBMCs (46). Consistent with the *in vitro* study, oral administration of SFN decreased mRNA levels of pro-inflammatory markers in PBMCs from ASD patients (46), conversely, increased the mRNA levels of cytoprotective enzymes (NQO1, HO-1, AKR1C1) and heat shock proteins (HSP27 and HSP70) (46). Besides, Bent et al. identified 77 urinary metabolites correlated with alterations in autism-like symptoms induced by SFN therapy, primarily in oxidative stress, amino acid/gut microbiome, neurotransmitters, hormones, and sphingomyelin metabolism (74). On this account, we can adopt urinary metabolomics analysis to identify how certain biological interventions affect specific symptoms of ASD, providing clues to reveal the pathophysiology of ASD. Furthermore, we can tailor treatment to each ASD individual's unique metabolomics profile in the future.

To sum up, we summarized the extensive evidence for the beneficial effects of SFN, extending from *in vitro* studies, to animal models, to various clinical studies. Based on the results from human studies, SFN was safe and well-tolerated without serious adverse effects.

### Curcumin

Curcumin, (1,7-bis(4-hydroxy-3-methoxyphenyl)-1,6-heptadiene-3,5-dione) along with its mono and dimethoxy derivatives, collectively called curcuminoids (75), which is a hydrophobic polyphenol derived from the rhizome of turmeric (Curcuma longa) (76). Curcumin can cross the blood-brain barrier and has shown to be neuroprotective through upregulating the Nrf2 gene.

Six studies explored the impacts of curcumin on autism-like behaviors and molecular alterations caused by different animal models of ASD. Firstly, curcumin treatment (50, 100, and 200 mg/kg for 4 weeks, orally) restored neurological, behavioral, biochemical, and molecular alterations induced by PPA in a dose-dependent manner (51). Specifically, curcumin improved autism-like behaviors (i.e., reciprocal social interactions, repetitive behaviors, stereotypes, locomotor activity, anxiety, depression, spatial learning, and memory), and suppressed oxidative nitrosative stress (i.e., restored lipid peroxidation, nitrite, GSH, superoxide dismutase, and catalase levels), mitochondrial dysfunction, TNF-α, and matrix metalloproteinases (MMP-9) levels (51). Similarly, neonatal curcumin treatment ameliorated autism-like symptoms of BTBR mice, including social deficits, repetitive behaviors, and cognitive impairments (47). Moreover, curcumin greatly alleviated the suppression of hippocampal neurogenesis in BTBR mice, resulting in increased neurogenesis and proliferation of neural progenitor cells (47). In the VPA model, curcumin also plays a significant role in attenuating abnormalities. On the one hand, curcumin significantly restored abnormal development and behavioral disorders (i.e., social communication and interaction, learning, and memory ability) in VPA rats (48). Besides, curcumin up-regulated the expression of neurons in hippocampal DG and CA3 regions and down-regulated the expression of astrocytes (48). On the other hand, Al-Askar et al. demonstrated that postnatal curcumin treatment reversed various impaired parameters (i.e., IFN-γ, serotonin, glutamine, GSH, glutathione S-transferase, lipid peroxidase, CYP450, IL-6, glutamate, and GSSG) in the VPA model (49). Especially, curcumin improved delayed maturation and abnormal weight (49). Lastly, Chen et al. demonstrated that curcumin ameliorated autistic behavior in a dose-dependent manner (52). Besides, postnatal curcumin treatment increased brain-derived neurotrophic factor (BNDF) level in the temporal cortex at a dose of 50 mg/kg, whereas no effect at a dose of 10 or 30 mg/kg (50, 52). Taken together, curcumin may be an effective option for ASD therapy. However, more clinical studies are needed to prove whether curcumin can be applied to humans.

### Agmatine

Agmatine is an endogenous biogenic polyamine synthesized from the decarboxylation of L-arginine in the brain (54). Like other neurotransmitters, agmatine is synthesized, stored, and released in the brain (77). Besides, agmatine could cross the blood-brain barrier (78). Most neurons containing agmatine were located in the hypothalamus, forebrain, and cerebral cortex (77), which played important roles in visceral and neuroendocrine control, pain perception, emotion, and cognition (77, 79). It was reported that agmatine could resist inflammation, apoptotic, and oxidant; could promote angiogenesis and neurogenic; could attenuate gliosis and edema (77, 80). Notably, studies evidenced the important role of agmatine in the pathogenesis of epilepsy, neurodegenerative disorders, and several psychiatric diseases (77, 81, 82). Interestingly, we found a significantly lower level of agmatine in patients with ASD compared with the control group (77), which proved that agmatine can be used as a target for ASD treatment.

Only one animal study investigated agmatine effects on ameliorating ASD symptoms using the VPA animal model. In the study by Kim et al., agmatine sulfate (25, 50, and 100 mg/kg) or saline was supplemented in the VPA model via intraperitoneal injection 30 min before each behavior test (54). As a result, the dose level over 50 mg/kg of agmatine, and not its metabolites, improved the social defect and repetitive and hyperactive behaviors, as well as decreasing seizure threshold in VPA rats. Besides, agmatine normalized the overactive ERK signaling in the prefrontal cortex and hippocampus of VPA-exposed rats (54). Taken together, it suggested a possible therapeutic role of agmatine in ameliorating autism-like symptoms in the VPA animal model.

### Naringenin

Naringenin (5,7-Dihydroxy-2-(4-hydroxy phenyl) chroman-4-one) is a flavanone abundantly found in grapefruit, orange, and tomato peels (53, 74). Naringenin exerts its antioxidant effect by inhibiting the NF-κB pathway to reduce oxidative damage to DNA by mice exposed to radiation (83). However, due to the low solubility and high metabolism of naringenin, its bioavailability before entering the systemic circulation is poor, which hinders its clinical efficacy (84).

One study investigated the pharmacotherapeutic potential of naringenin and its brain targeted nanoformulations in ASD. In this study, naringenin (25, 50, and 100 mg/kg), uncoated and coated (GSH & Tween-80) naringenin loaded poly (lactic-co-glycolic acid) (PLGA) nanoparticles (25 mg/kg), and minocycline (50 mg/kg) were given to PPA-induced rats orally for 29 days (53). As a result, in the unencapsulated form, naringenin was only effective at a higher dose of 100 mg/kg because it cannot cross the blood-brain barriers with low bioavailability and P-glycoprotein efflux. Conversely, GSH and Tween-80 coated naringenin nanocarriers acted as multifactorial neurotherapeutic agents, restoring abnormal neuropathological manifestations in the PPA model (53). Taken together, Bhandari et al. demonstrated that PLGA nanoparticles can not only improve naringenin's bioavailability but also enhance their brain absorption capacity through coating with ligands such as GSH and Tween-80, which is an effective delivery system (53). Therefore, naringenin and its coated nanocarriers showed strong clinical potential in attenuating neuro-psychopathological abnormalities associated with ASD.

## Discussion

To the best of our knowledge, this is the first systematic review to qualitatively synthesize existing evidence on the effects of Nrf2 activators administration on ASD *in vitro* studies, animal studies, and clinical studies. Ultimately, we identified five Nrf2 activators associated with ASD therapy, including RSV, SFN, curcumin, agmatine, and naringenin. We found that the administration of Nrf2 activators could improve autism-like symptoms and molecular alterations in ASD by against inflammation/immune dysfunction, oxidative stress, and mitochondrial dysfunction. Although these five Nrf2 activators have the potential to improve ASD symptoms, they were based on different mechanisms related to Nrf2, as each has unique metabolic patterns and biological activities.

In our systematic review, although a large number of preclinical studies and a small number of clinical studies have proved the efficacy and safety of Nrf2 activators in the treatment of ASD, caution should be warranted in attempting to extrapolate their effects in human studies. If Nrf2 activators were used in high concentrations in tissues lacking Nrf2, there was even a potential risk that Nrf2 may be harmful to subjects. Strikingly, phytochemicals present in fruits and vegetables can promote activation and protection of Nrf2 at low concentration, while would cause toxicity at high doses (85). In addition to the dosage of the Nrf2 activator, the duration of its use should also be considered. To illustrate, Nrf2 activation can reduce tumorigenesis in the early stage, while it promotes the development of treatment-resistant cancer cells in the later stage (20). RSV was well-tolerated without severe side effects in our review. However, in our systematic review, the observation period of the RSV clinical research was only 10 weeks (55), which may not represent the long-term efficacy and side effects of RSV treatment. Instead, RSV supplementation in pregnant non-human primates may cause abnormalities in the fetus (86), consistent with the view that RSV is a teratogen (34). Besides, in one clinical trial of patients with multiple myeloma, RSV treatment has caused various serious adverse events, especially renal failure (87). Therefore, RSV is generally only used as a potential treatment to elucidate the etiology and pathophysiology of ASD. Similarly, while curcumin has been recognized to be safe by the US Food and Drug Administration, curcumin shows conflicting effects on genotoxicity and anti-genotoxicity. Curcumin as low as 0.1 mM may cause disproportionate DNA isolation, nuclear disruption, and micronucleation in proliferating endothelial cells (88). It was also reported that taking curcumin 0.2 mg/kg body weight daily increased the incidence of labial adenoma, hepatocellular adenoma, small intestine cancer, and hepatocellular adenoma in rodents (89). As for SFN, although it was generally safe and well-tolerated in animal models and humans, several side effects were found (i.e., insomnia, vomiting, flatulence, diarrhea, etc.). Interestingly, researchers showed that the dose-response effect of Nrf2 activators may be reversed in pathological tissues, that is, normal beneficial concentrations of phytochemicals will produce toxic effects. For instance, Fragoulis et al. demonstrated the opposite effects of SFN on naïve and TNF-α-stimulated synoviocytes (90). Namely, SFN induced apoptosis of inflammatory synovial cells by activating Nrf2 in naïve synovial cells. Taken together, despite the beneficial effects and few adverse effects of Nrf2 activators, it is necessary to balance their concentration and duration to elucidate their applicability in clinical trials.

Several animal models have been used in this systematic review, including four pharmacological models and one genetic model. VPA, a short-chain fatty acid, is widely used as an antiepileptic drug and mood stabilizer (28). This model is derived from an epidemiological study in which mothers exposed to VPA during pregnancy have a higher risk of ASD among their offspring (91). PPA is an endogenous short-chain fatty acid that can cross the blood-brain barrier (24). Moreover, PPA causes alterations in serotonin, dopamine, and glutamate levels by stimulating calcium release, which simulates what occurs in autism (92, 93). Indeed, numerous studies evidenced higher levels of PPA and other intestinal short-chain fatty acids in ASD subjects (28). The BTBR is the only available genetic model in ASD preclinical studies to date (94), initially used to study insulin resistance, diabetes-induced nephropathy, and phenylketonuria (95). BTBR mice are inbred strains, exhibiting social deficits and repetitive behaviors (96) as well as several immune abnormalities (97), similar to the core symptoms and molecular defects of ASD (35). Thus, the BTBR model is currently a good choice for studying the pathogenesis of ASD. Besides, exposure of pregnant women to any form of progestins (i.e., oral contraceptives, food, beverages, etc.) has been shown to increase the risk of ASD in their offspring (24). Generally, the first trimester of human pregnancy was considered the most sensitive period for prenatal exposure to risk factors. Accordingly, exposure to progestin in the first 7 days of pregnancy in the animal study may be more reasonable. However, prenatal progestin exposure occurred throughout the gestation of Sprague-Dawley rats in the study by Xie et al. (41), which rarely occurs in humans.

Except for agmatine, the Nrf2 activators identified in this systematic review were natural dietary phytochemicals. Mostly, their bioavailability depends on their chemical structure and mode of administration. In particular, due to their first-pass metabolism, oral bioavailability is low (98, 99). After oral administration, partial Nrf2 activators exhibited poor absorption and undergone fast biotransformation, with fewer compounds entering the systemic circulation (89). Based on this, higher doses were required to achieve therapeutic effects. However, for safety reasons, it is necessary to adjust drugs' concentrations to understand their applicability in clinical trials. To date, researchers have developed various strategies to improve the bioavailability of these drugs, thereby achieving therapeutic and safety effects in several neurodegenerative diseases (24). For example, the low oral bioavailability of curcumin hinders its development as a therapeutic drug and/or functional food. However, the animal pharmacokinetic study has shown that the bioavailability of amorphous solid dispersion of curcumin was up to ~13-fold higher than that of unformulated curcumin. Moreover, amorphous curcumin solid dispersions showed enhanced anti-inflammatory activity even at doses 10-fold lower than unconfigured curcumin (75). Besides, nanotechnology has been applied to prepare curcumin nano preparations with higher solubility and slower biotransformation rate, thereby enhancing its bioavailability. There have been several nanoformulations of curcumin including phospholipid complexes, emulsion-based delivery systems, liposomes, polymeric micelles, and curcumin nanoparticle (89). In this systematic review, Bhandari et al. demonstrated that PLGA nanoparticles can not only improve naringenin's bioavailability but also enhance their brain absorption capacity through coating with ligands such as GSH and Tween-80, which was an effective delivery system (53). In conclusion, naringenin and its coated nanocarriers have strong clinical potential in attenuating neuro-psychopathology associated with ASD. However, except for naringenin, there is currently a lack of research on targeted drug delivery systems for curcumin, RSV, SFN, and agmatine. Hence, while studying Nrf2 activators in the treatment of ASD, we should also develop a suitable drug delivery system to enhance their therapeutic potential. In this way, it will not only overcome the low bioavailability issues but also show sustained effects at a lower dose, thereby improving patient compliance.

This systematic review has several limitations. Firstly, this review has some limitations in research design. Different studies differ in research objects (species, age, and gender), intervention methods (drug type, drug dosage, administration method, administration frequency, and duration), outcome evaluation methods, etc., which may lead to inter-study heterogeneity. In terms of experimental subjects, this review covered animals (rats and mice), cells, and humans. Species differences might account for some of the variability of results concerning the efficacy of the various agents. Furthermore, there may be bioavailability differences and metabolism differences as well (inactive, active, toxic metabolites). Therefore, it is difficult to predict whether the results found in animals of different species could be applied to humans. Second, there are few studies to support the Nrf2 activators as dietary phytochemicals in ASD, even though several preclinical studies. Perhaps, these mixed results may be attributed to imperfect statistical analyses, small sample size, and inadequate study design. Therefore, caution should be warranted in attempting to extrapolate their effects in human studies. Third, most preclinical studies involved mainly male animals, and even in clinical studies mainly male patients. It was well-known that ASD affected males more than females by a ratio of 4:1 (28). Fourth, we excluded studies that were not published in English and Chinese, which may lead to eliminating potentially relevant studies.

ASD was considered as a heterogeneous disorder. Patients with ASD will show diverse efficacy in response to the same treatment and intervention (28). Therefore, further RCTs should focus on assessing the efficacy of interventions targeting specific subgroups of ASD. a different subgroup of ASD patients besides evaluating the monotherapy. Moreover, the researchers used animal models of ASD in which animals were prenatal exposure to large doses of progestin, VPA, and PPA to simulate long-term low dose exposure to them in humans, which may limit application in humans. Moreover, despite several Nrf2 activators showed fine efficacy *in vitro* studies, their low bioavailability limits their activities in the target tissues *in vivo* studies (60). Consequently, researchers must carefully interpret *in vitro* studies when attempting to infer the roles of *in vivo* studies. Most importantly, most Nrf2 activators discussed in our review have multiple functions and molecular targets, therefore, pharmacological research still cannot completely replace the genetic approach. Moreover, due to the difference in animal models and research species, it is not reliable to extrapolate results from one environment to the other. Taken together, further research should improve the standardization of study populations/model, dosage, and outcomes measured, thereby contributed to effective cross-comparisons and meta-analyses of study outcomes. Because of the complex alterations of ASD, studying the pathogenesis of ASD and determining an appropriate therapeutic target remains a formidable challenge in overcoming ASD.

## Conclusion

Our systematic review provided suggestive evidence that Nrf2 activators have a potentially beneficial role in improving autism-like behaviors and abnormal molecular alterations through oxidant stress, inflammation, and mitochondrial dysfunction. To sum up, the Nrf2 activators (i.e., RSV, SFN, curcumin, agmatine, and naringenin) identified in this systematic review opened up a new approach to ASD treatment. While these Nrf2 activators are regarded as natural diets to treat ASD and are considered to be relatively safer and healthier methods for humans, they are still limited to becoming a standard treatment because of lacking sufficient proof of their effectiveness and safety. Accordingly, caution should be warranted in attempting to extrapolate their effects in human studies, and better design and more rigorous research are required before they can be determined as a therapeutic option. In a word, Nrf2-based therapy is still an open field issue, and when applied, we should consider a more preventative approach in the future.

## Data Availability Statement

The original contributions presented in the study are included in the article/supplementary material, further inquiries can be directed to the corresponding author/s.

## Author Contributions

JY and YL conceived the manuscript idea. JY and XF researched the manuscript and identified eligible studies. JY, XF, and XL extracted data and performed the quality appraisal of studies. YL was responsible for funding acquisition. All authors critically reviewed the manuscript and read and agree to the published version of the manuscript.

## Conflict of Interest

The authors declare that the research was conducted in the absence of any commercial or financial relationships that could be construed as a potential conflict of interest.
